# Addition of a Second Calcaneal Pin for Spanning Ankle External Fixation

**DOI:** 10.7759/cureus.55312

**Published:** 2024-03-01

**Authors:** Meridith Deluca, Brett A Hoffman, Kevin Serdahely, Sreeram Ravi, Christopher Sanford

**Affiliations:** 1 Orthopedics, The University of Toledo College of Medicine and Life Sciences, Toledo, USA; 2 Orthopedics, Penn State Health Milton S. Hershey Medical Center, State College, USA

**Keywords:** technique, fracture, calcaneus, ankle, external fixation

## Abstract

Spanning ankle external fixation is a commonly used technique for the treatment of fractures of the lower extremity. Traditionally, a single pin is placed in the safe zone of the calcaneus to provide a point of traction for fracture reduction and stabilization. Complications include infection and pin loosening with subsequent loss of fracture reduction. We aim to highlight the benefits and techniques of adding a second calcaneal pin to reduce the likelihood of infection, pin loosening, and possible loss of fracture reduction. Using the standard medial-to-lateral placement technique, two centrally threaded Schanz pins were placed within the safe zone of the calcaneus approximately 2 cm apart and were connected by clamps and a short carbon fiber rod. The remainder of the external fixation apparatus is assembled using a standard technique after obtaining fracture reduction. There is an increased incidence of infection and pin loosening with decreased bone quality and a longer duration within an external fixator. The addition of a second calcaneal pin can be used to reduce the incidence of pin loosening and associated sequela, especially in patients with decreased bone quality, thus improving outcomes for patients undergoing spanning ankle external fixation.

## Introduction

Spanning external fixation of the ankle joint is a powerful tool for temporary stabilization of injuries that require rigid stabilization while allowing access to the soft tissue overlying the fracture site, minimizing edema, and muscular atrophy [1,2]. External fixation remains indicated for primary stabilization in patients experiencing multiple traumas. It is also indicated for patients with severe soft tissue injuries not allowing for early definitive fixation or for patients generally inappropriate for prolonged surgery. The benefits of this method involve its ease of application and minimal impact on surrounding tissues [1]. These benefits extend to both provisional and definitive fixation as the technique is relatively simple with few absolute contraindications [3].

Complications of external fixation are most frequently associated with pin sites. Pin site infection is the most common complication, with reported infection rates up to 85% [4]. As pins traverse the skin, their external components provide a potential nidus for infection. A vast majority of cases involving pin site infection are resolved with oral and/or topical antibiotics and pin care [5]. There are several additional factors that may increase the rate of infection which include smoking, drug use, obesity, diabetes, and poor bone quality [4,6,7]. These typically are the same factors that lead to poor wound healing and bone healing [4,6,7]. Studies have demonstrated a significant reduction of pin tract infections when topical agents were used prophylactically in pin care protocols [4].

Fracture at the site of pin insertion is another complication that may arise during treatment. Improper placement of pins in the calcaneus may cause an avulsion fracture if inserted too posteriorly [8,9]. Additional fracture risk is present upon pin removal, as the empty pin tract may reduce the compressive strength of the bone. As with other pain-related complications, fracture risk is increased in patients with comorbid conditions that may delay healing [4].

Pin loosening is perhaps the most important consideration, as it can lead to both infection and fracture at the pin site as well as loss of fracture reduction. A loose pin can arise from repeated loading forces and torque. The complication of pin loosening can compromise fracture reduction and lead to further soft tissue compromise. These complications are amplified in populations at risk for increased infection and poor bone healing [5]. Centrally threaded calcaneal pins are typically used with the strength of this fixation dependent upon the quality and strength of the surrounding bone [10]. Previous trials of pins with a variety of pin coatings to promote bone healing and improve pin osseointegration have been conducted [4,10]. Some data indicate that uncoated pins may be less appropriate for use in the tibial metaphysis, as these pins tend to loosen before removal [10]. However, there is not sufficient evidence to support the preferential use of coated pins, and the significant increase in cost for such pins may prove to be prohibitively expensive [10-12].

Anatomic safe zone

When placing percutaneous pins in the calcaneus, consideration must be made to avoid damage to local structures. With the tibial neurovascular bundle near the point of insertion, many studies have been done to define a relatively safe zone within which damage is unlikely to occur [13]. The anatomic safe zone is defined as a 3.1 cm radius approximately one-fourth to one-third of the distance between the posteroinferior calcaneus and the inferior tip of the medial malleolus [8,13]. Even in the presence of edema, bony landmarks remain palpable for identification of this safe zone [9]. The use of intraoperative fluoroscopy is also routinely used to confirm appropriate pin placement. This safe area has been proven in cadaveric studies to be relatively devoid of important neurovascular structures and is the preferred region in which to insert calcaneal pins for spanning ankle external fixation [9,14]. The primary structure at risk during placement of calcaneal pins is the medial calcaneal nerve, which varies in its branching pattern from the tibial nerve. The posterior branches of this nerve are most vulnerable, a risk that can be mitigated by more posterior placement of pins. However, the incidence of avulsion fractures increases as pin placement moves farther posterior. Despite the risk of nerve injury and the possibility of sensory sequelae, this remains the safest area for placement of calcaneal pins during spanning ankle external fixation [9].

Given that the most frequent complications associated with external fixators pertain to the pins, extensive research has been conducted to lower the incidence of pin site complications. Here, we present a variation on pin placement that the authors have found to be beneficial in certain patient populations. We describe the insertion of a second pin in the anatomic safe zone of the calcaneus. This technique aims to reduce the net force on each pin with a subsequent reduction in the incidence of pin loosening, pin-tract infection, and calcaneal fracture.

## Technical report

**Figure 1:**
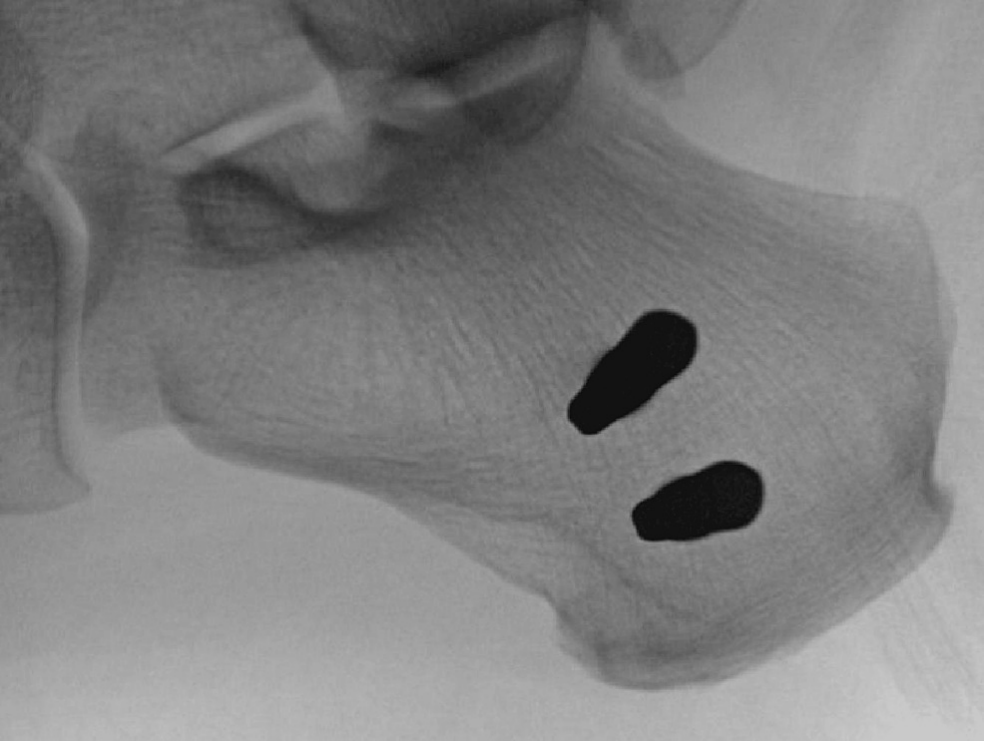
Lateral view of the dual calcaneal pin placement within the anatomic safe zone.

**Figure 2:**
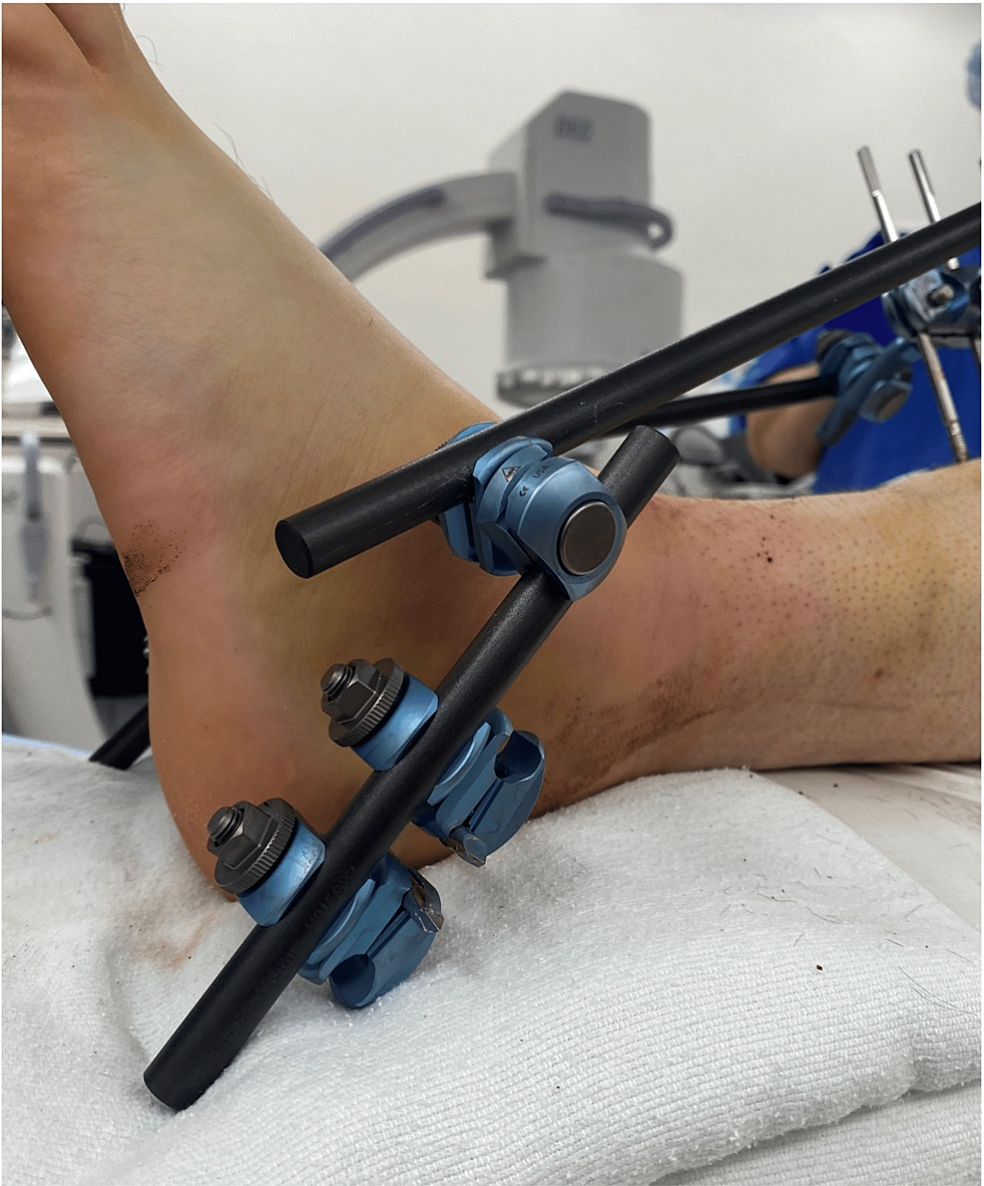
External fixation apparatus.

A 1-cm longitudinal incision is made over the medial calcaneal safe zone and spread to the bone. A centrally threaded 5.0 mm Schanz pin is then inserted perpendicular to the medial cortex and parallel to the axial plane of the calcaneus until the threads are fully seated within the bone and there is a roughly equal pin exposed medially and laterally. As shown in Figure 1, a second pin is then placed parallel approximately 2 cm proximal and anterior to the first pin. Each pin is placed using one small incision. Figure 2 shows the external fixation apparatus.

**Figure 3:**
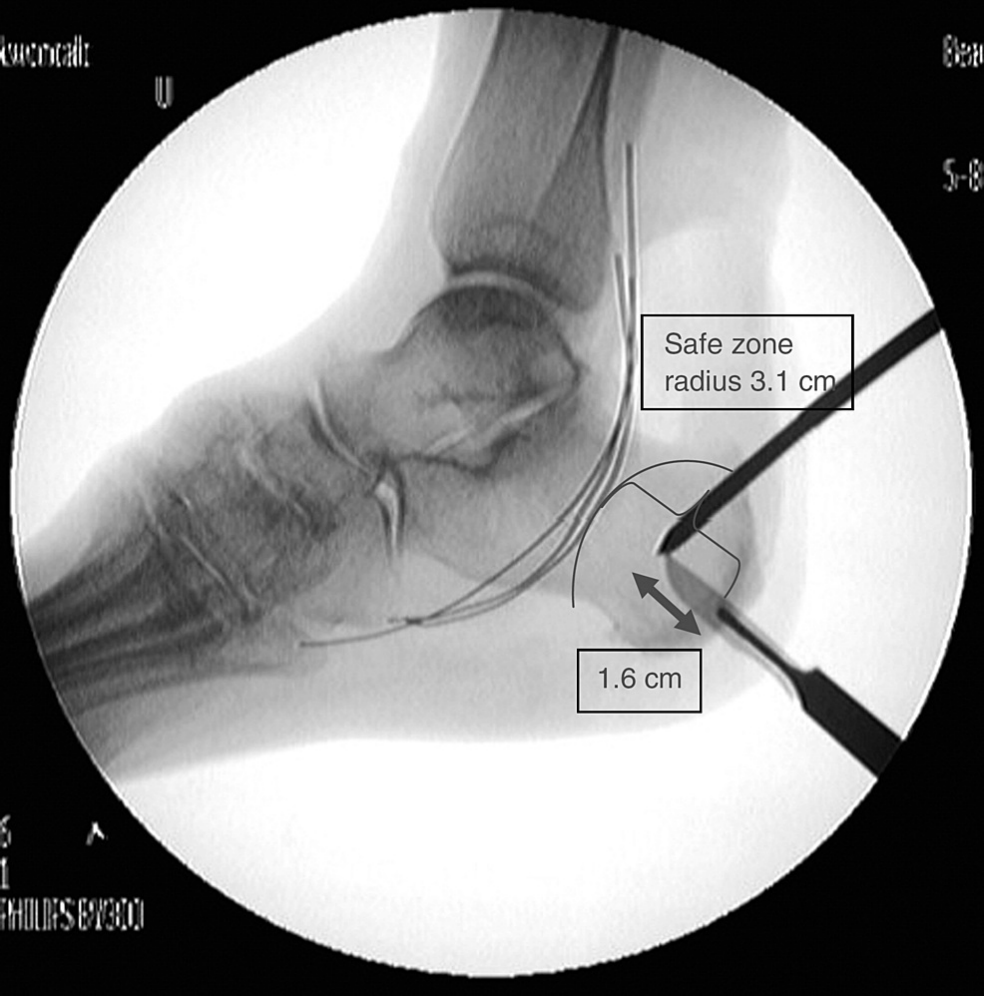
Radiographic image of the anatomic safe zone.

Both pins are placed within the safe zone of the calcaneus. Figure 3 shows the anatomic safe zone as described by Kwon et al. [13]. These two calcaneal pins are connected by clamps and a short carbon fiber rod. The remainder of the external fixator is assembled using standard technique.

## Discussion

Considering the high incidence of pin site complications during the use of external fixation devices, we present a technique variation that may prove beneficial in some patient populations. With this study, we aim to describe a safe technique for adding a second calcaneal pin to an ankle-spanning external fixator construct. This technique provides increased stability while reducing the likelihood of pin loosening and negative sequelae. Some surgeons attempt to increase stability with the addition of an additional pin through the base of the first and second metatarsals. However, this technique carries significant risk as it has been shown to injure the clinically significant deep plantar branch of the dorsalis pedis artery [15]. With the addition of a second calcaneal pin, we expect to see a reduction in the incidence of pin loosening which could occur through two mechanisms. First, the addition of a second pin reduces the individual net rotational torque per pin, thus increasing the total force required to cause loosening. Second, the addition of a second pin changes the force vector applied by the external fixator. Instead of a single distal point of traction with force applied longitudinally along the axis of the fixator, the forces are distributed slightly off the axis of the tibia. This could also provide extra stress to the bone between the two pins, thereby increasing the friction force exerted on the second pin. 

A consideration that can affect the use of multiple pins in close proximity is the increased fracture risk from the compressive forces of the additional pin as well as the increased local stress on the bone between the two pins. There is currently no literature addressing the fracture risk of placing two Schantz pins in the anatomic safe zone of the calcaneus in the coronal plane. Cadaveric studies have assessed the viability of anteromedial pin placement in the sustentaculum tali, but edema frequently obscures this landmark from palpation. Additionally, the risk of entering the subtalar joint or injuring the adjacent tendons during pin placement outweighs the potential benefits of this technique [8]. Research has shown that both high-quality as well as osteopenic bone can handle the stress of a second pin with little fracture risk during manipulation and after the removal of the pins [16]. It is worth noting that this technique has been performed 20 times by the authors with no incidents of pin site fracture.

In prior cadaveric studies, it was shown that a single 6 mm pin removed from calcaneal samples reduced the compressive strength of the calcaneus by 22% [17]. This locally increased stress around the pin tract can lead to an increased rate of fracture on load bearing in the weeks after removal. This is a factor to be considered with the addition of a second pin. Brooks et al. showed that these factors may be smaller in the cancellous bone which comprises a large proportion of the calcaneus [17]. The addition of a second pin is designed to mitigate the catastrophic effect that a loose calcaneal pin can have on calcaneal compressive strength if it is to toggle and bore a large hole in the calcaneus. There is currently no literature dedicated to studying the impact of a second pin on calcaneal compressive strength, however, putting multiple tracts in a localized area of bone is commonplace in orthopedics. The authors feel that the stability added by the second pin outweighs any decrease in calcaneal compressive strength and would like to again reference their experience of 20 cases without any fracture-related complications.

In their study on the effects of bone properties on pin loosening, Donaldson et al. stated that bone yielding is a problem that is experienced when metal implants interface with bone [18]. In this situation, the bone surrounding the metal implant is subjected to higher stress than other areas and can promote a loss of bone integrity, especially in lower-quality bone specimens [18]. Research demonstrates that it is possible to identify a patient population that is at increased risk for pin loosening based on their physiologic factors as well as the proposed pin material [4]. This type of predictive modeling may help identify patients who would be ideal candidates to receive a second calcaneal pin so that loosening can be prevented. 

Another important consideration is the incidence of pin-tract infections and deep infections such as osteomyelitis and pin-tract sepsis. When using external fixation, superficial pin-tract infections are expected and are easily treated with debridement, antibiotics, and pin removal when necessary. It is possible that the addition of a second calcaneal pin will increase the rate of superficial pin-tract infection. While these infections do not significantly impact morbidity, the same cannot be said for their sequela. Deep infections like osteomyelitis are difficult to treat, especially in the calcaneus, and drastically increase morbidity [19]. These infections often occur when loosening at the pin-bone interface provides a superficial pin-tract infection with the necessary access to colonize and infect the bone [20]. The additional biomechanical stability provided by the second calcaneal pin reduces the risk of pin loosening and should thus reduce the incidence of deep infections such as osteomyelitis. While greater patient volumes will be needed to truly evaluate the effect that a second calcaneal pin will have on infection rate, the authors have had no deep or superficial infections in their 20 cases.

## Conclusions

There is an increased incidence of infection and pin loosening in patients with decreased bone quality and a longer duration within an external fixator for patients undergoing spanning ankle external fixation. A second calcaneal pin offers an alternative to traditional spanning-ankle fixation which can reduce rates of pin loosening and the associated sequela. Future studies will aim to evaluate both the failure parameters of a second calcaneal pin as well as the effect of a second calcaneal pin on the compressive strength of the calcaneus in cadaveric specimens. These studies would seek to gain insight into the changes in loading and compressive forces in the presence of a second pin.
